# Efficient Acceleration of Stencil Applications through In-Memory Computing

**DOI:** 10.3390/mi11060622

**Published:** 2020-06-26

**Authors:** Hasan Erdem Yantır, Ahmed M. Eltawil, Khaled N. Salama

**Affiliations:** Computer, Electrical and Mathematical Sciences and Engineering Division, King Abdullah University of Science and Technology (KAUST), Thuwal 23955-6900, Saudi Arabia; ahmed.eltawil@kaust.edu.sa

**Keywords:** stencil codes, Laplace, Jacobi iteration, memristor, in-memory computing, associative processors, image processing, single instruction, multiple data

## Abstract

The traditional computer architectures severely suffer from the bottleneck between the processing elements and memory that is the biggest barrier in front of their scalability. Nevertheless, the amount of data that applications need to process is increasing rapidly, especially after the era of big data and artificial intelligence. This fact forces new constraints in computer architecture design towards more data-centric principles. Therefore, new paradigms such as in-memory and near-memory processors have begun to emerge to counteract the memory bottleneck by bringing memory closer to computation or integrating them. Associative processors are a promising candidate for in-memory computation, which combines the processor and memory in the same location to alleviate the memory bottleneck. One of the applications that need iterative processing of a huge amount of data is stencil codes. Considering this feature, associative processors can provide a paramount advantage for stencil codes. For demonstration, two in-memory associative processor architectures for 2D stencil codes are proposed, implemented by both emerging memristor and traditional SRAM technologies. The proposed architecture achieves a promising efficiency for a variety of stencil applications and thus proves its applicability for scientific stencil computing.

## 1. Introduction

The limitations of traditional computer architectures have become explicit as the industry reaches to the end of Dennard scaling [1] and Moore’s law [2] where the data movement is dominated over both overall system energy and performance. More than 90% of the energy consumed by an instruction is spent on memory access [3]. Considering the current station in which 90% of the overall data has been produced in the last two years, which corresponds to a 9× increase in the total amount [4], the computer architectures responsible in the processing of all these data must be optimized in terms of data handling methodology. Certainly, the most important domain that needs such a massive amount of data is signal processing. The emergence of artificial intelligence (AI) and big data has dramatically increased the importance of signal processing since the raw data must be processed to obtain better accuracy achievement. On the other hand, there is no corresponding development in computer architectures to handle such an enormous amount of data at the same rate. Excessive increase in the amount of data to be processed and increasing complexity of computational tasks force the researchers towards more data-centric architectures rather than today’s processor-centric ones. One such application that highly requires data-centric computational platforms is stencil codes that are used in many computational domains [5,6].

The bottleneck of the current systems is generally caused by the communication between processor and memory. The memory systems cannot supply the data to the processor at the required processing rate. Moreover, the energy consumption spent on data access is an order of magnitude higher than the computation cost due to the out of chip access [3,7]. The ideal solution is combining processor and memory at the same location to alleviate the limited connection link between them. For this reason, there are recently many research attempts aiming either bringing the processor near the memory (i.e., near-memory computing) [8] or integrating them (i.e., in-memory computing) [9,10]. In-memory computation architectures are very diverse, ranging from analog computation by using the non-volatile memories [11,12,13,14] through the in-DRAM processing between the DRAM rows [15]. Among them, associative processors (APs) propose an applicable solution that performs the noise-free digital computation through the binary memory devices (e.g., memristor, SRAM, STT-RAM) [16,17]. Associative processors can be considered as a type of single instruction multiple data (SIMD) processor that combines the functionalities of processor and memory in the same location [16]. In AP, the operations are performed directly on the data residing in memory without moving them. Each memory row behaves as an individual processor together with its own special set of registers. Since an operation can be performed on all memory words in parallel, the execution time of operations does not depend on the vector size. This feature solves the memory-wall problem of traditional von Neumann architectures since there is no inter-dependence between memory and processor [18]. Even though the inherent latency of associative processors is much higher than the traditional architectures, it can result in better throughput and energy efficiency if the required degree of parallelism is demonstrated by the application [19]. In applications characterized by data parallelism, associative processors (APs) accomplish a remarkable acceleration [20], and can be employed as an accelerator near the main processor [21].

Stencil codes are a class of iterative kernels which update a given array (generally 2D or 3D) with respect to a specific pattern [22]. This pattern is called as a stencil. The code performs a sequence of iterations through a given array. In each iteration, all the elements of the arrays (i.e., cells) are updated. Stencil computations are highly used in the scientific computation domain for many purposes, including image processing, solving differential equations, computational fluid dynamics simulations (e.g., weather prediction), etc. Due to its importance, there are many studies in the literature that aims to propose an efficient architecture implementation for stencil codes [23,24,25]. Most of the studies are headed towards to field-programmable gate arrays (FPGAs) or graphical processing units (GPUs) based implementations since traditional central processing unit (CPU)-based solutions cannot fulfill the parallel processing requirements. As an example, the study in [26] proposes a GPU-based 2D stencil implementation using CUDA. The implementation exploits the multi-threading and optimizes the shared memory usage in GPUs. In [27], OpenCL implementation of four 3D stencil computations is proposed for GPU architectures, which exhibits superior performance than CUDA-based alternatives. In [28], a multi-core CPU based implementation is proposed together with the corresponding software optimization. In [29], an OpenCL-based FPGA implementation of some stencil codes is proposed in which a high-level synthesis language is used to generate the stencil codes. Similarly, in [30], a custom optimized high-level synthesis flow is presented for both area and throughput optimization. The FPGA-based approaches can be considered as near-memory architecture where the memory bottleneck problem is mitigated through the distributed internal memory inside the FPGA fabric. The study in [31] proposes a multi FPGA-based stencil implementation. The study in [32] proposes a parameterizable, generic VHDL template for parallel 2D stencil code applications on FPGAs instead of high-level synthesis solutions. In FPGA-based solutions, the performance is limited by both memory bandwidth and the amount of internal memory and logical resources inside the FPGA. After reaching their limits, increasing the parallelism does not increase the performance. The same rule also applies to GPU and CPU based implementations as well. Therefore these architectures limit the degree of parallelism to the number of cores that can be fit in a given chip area and available energy budgets. Considering the case that size and quality of the data are increasing rapidly, it is obvious that there is a need for more efficient domain-specific processor architectures to manage an enormous amount of data for stencil codes as pointed by the computational trends for beyond the Moore’s Law and Dennard Scaling [33].

The stencil computation generally requires basic operational complexity (i.e., a sum of weighted products), but large external memory bandwidth [26,29]. This is due to that it requires a number of accesses to the memory while updating each point. Therefore, most implementations of stencil code on traditional architectures suffer from bandwidth limitations [26,34]. As a promising solution, associative in-memory processors take advantage of content addressable memories, which provides an area-efficient, in-memory processing solution by integrating the computation and storage. In in-memory solutions, memory bandwidth can be considered as the amount of whole memory. For this reason, this study proposes a 2D stencil kernel architecture based on associative in-memory processing to eliminate the memory bottleneck. The study shows the two implementations by using both SRAMs and memristors. Since stencil codes are memory bound (i.e., the ratio of memory access to computation is high), APs provide a good processing environment for them. Furthermore, the implementation provides a considerable amount of energy savings and speedups in the system through approximate computing at some reasonable level. The rest of the study is organized as follows: In the following section, the background knowledge of both associative processors and stencil codes is presented. Section 3 introduces the proposed accelerator architecture in detail. Experimentation and evaluation results are discussed in Section 4. The final section concludes the work.

## 2. Background

### 2.1. Associate Processor

**Figure 1:**
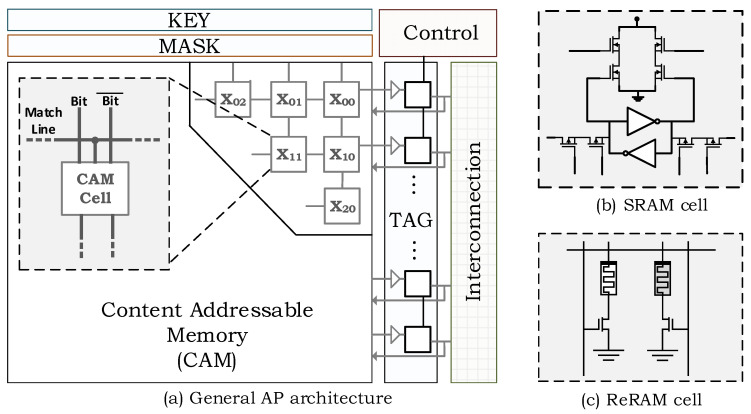
Architecture of an associative in-memory processor with SRAM and ReRAM based cell types.

Almost all computer architectures use traditional Boolean logic to perform logical and arithmetic operations. On the other hand, there are many other techniques as well to perform the operations non traditionally. Associative computing is one of them that exploits the associativity principles of memories for logical and arithmetic computations. The architecture of an associative processor (AP) is presented in Figure 1, which consists of a content addressable memory (CAM), controller, interconnection circuit, and some specific registers (key, mask, and tag). The CAM stores the data on which operations are performed. The key and mask registers are used to search a given data inside the specified columns of the CAM. The key register keeps the data to be searched inside the CAM. The mask register points the specified column locations. The tag registers are used to keep track of row locations that have the searched data. Therefore, each row has its own single bit tag register even though mask and key registers are common for all CAM rows. The controller generates the instructions (key and mask pairs) for the corresponding operation (e.g., addition, subtraction, etc.) and checks the tag bits to carry on the operations. The rows tagged with logic-1 means that the corresponding CAM row has been matched with the given key and mask value. For example, if the key is set as 101 and mask as 011, the tag bits of the corresponding rows whose first and seconds bits are logic-1 and logic-0 respectively become logic-1. The third bit is not searched for logic-0 since its corresponding mask bit is logic-0 (i.e., not activated). The interconnection matrix is a basic circuit-switched matrix which used to communicate with other APs as column parallel fashion. The architecture can also incorporate low-power mechanisms such as selective compare, where within a lookup table (LUT) pass, the matched rows are not precharged again since it is not possible to get another match in this row [35]. As the most important part of the CAM array, the cells can either be implemented by traditional SRAM memory or alternatively by emerging non-volatile memories such as memristor (ReRAM) or STT-RAM. This study shows the two implementation candidates for APs, which are SRAM-based and ReRAM-based. Figure 1 shows the corresponding cell implementations. The traditional NOR-type CAM cell is used for SRAM-based implementation [36]. ReRAM-based implementations exploit the two-transistor, two-memristor ternary CAM cell structure, as studied in [37]. In both of the implementations, the functionally is performed exactly as same, but there are some trade-offs between them. For example, ReRAM-based implementation minimizes the static power consumption since the cells are non-volatile [38], but requires higher energy consumption during the write operation. On the other hand, SRAM-based implementation suffers from static energy consumption which becomes more severe as process technology improves, but provides low-cost write and less delay.

In traditional processor architectures, the data are sent over the functionality. In other words, the data are read from the main memory and sent to the processor to perform the operations on them. On the contrary, in associative processing, the operands stay inside the processor (i.e., in memory), and the functionality is sent over the data. Therefore, operations are performed inside the memory as in-place without moving them. An operation on AP is carried out by consecutive compare and write phases. During the compare phase, the content is selected inside the memory, and in the write phase, the corresponding functionality is applied to the selected rows which hold the corresponding data. Depending on the desired arithmetic operation, the controller sets the mask and key values by referencing a lookup table (LUT) for compare and write operations. The following example clarifies the in-place addition operation on AP.

**Figure 2:**
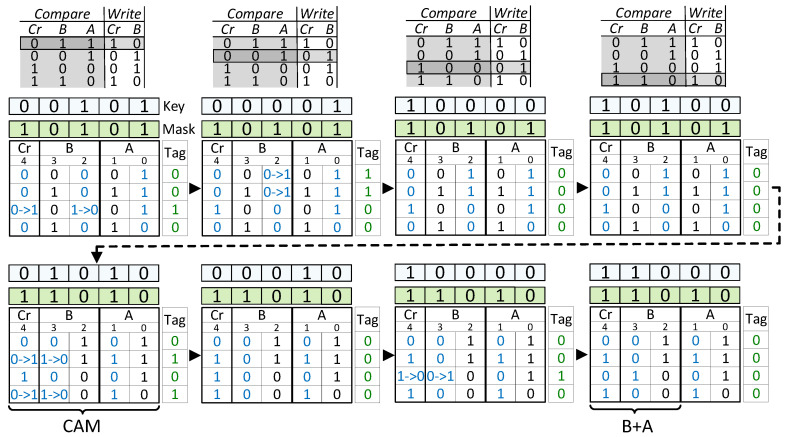
The sequence of compare and write operations are shown for a complete vector addition operation on 2-bit, 4 × 1 vector pairs of A (column 1-0), and B (column 3-2). The highlighted lookup table (LUT) entry shows the applied key values to the corresponding content addressable memory (CAM) columns specified by the mask register, and the arrows indicate the flow.

Figure 2 illustrates the complete flow for in-place addition of two 4×1 2-bit signed vectors, A and B, i.e., B[i]←B[i]+A[i],i=0…3, where the tables in the first row correspond to in-place addition LUT, and the others show the progress in the CAM content together with the key/mask values and the tag status. Initially, A contains (i.e., columns 1-0) the values of [1; −1; 1; −2] and B (i.e., columns 3-2) contains the values of [0; −2; 1; −2] in binary 2’s complement. Cr (carry) column (i.e., column 4) is initially all 0 s. In LUT, the highlighted entry shows the applied key on the masked columns. Each entry corresponds to a combination of different Cr, B, A. Even though there is a total of eight ($2^{3}$) combinations, only four of them are used since others have no effect on the operation [35]. In each CAM of the figure, the key value from the compare column of the LUT is searched in the masked columns of the CAM. The arrows specify the flow of the operation. In the first row, partial addition operation is performed on the first bits of A and B while Cr holds the carry. Therefore, the mask register is set as logic-1 for Cr and the first columns of A and B. The second row similarly corresponds to addition on second bits. After each comparison, the matching rows are tagged with logic-1, as indicated in its vertical tag register. Then, corresponding LUT entry (shown in the write column of the LUT) is written only to the masked cells in the rows whose tag register is logic-1. For example, in the first table, “011” is searched in Cr, B, and A columns, respectively. The third-row matches by indicating a logic-1 in its tag register. As a result, logic-1 is written to the Cr column, and logic-0 is written for the B column. Normally, this operation represents a full adder for the combination of 0 + 1 + 1, where the result is logic-0 and carry is logic-1 (“10” as together). By applying all combinations of inputs on each bit locations, column-wise full addition is performed. The process is repeated for all the passes in the prescribed order shown in Figure 2. Finally, the value stored in Cr and B becomes [1; −3; 2; −4] which is equal to B+A (i.e., [0 + 1; −2 + −1; 1 + 1; −2 + −2]). In general, adding two vectors that are *m*-bit wide takes 8m cycles (4m compares and 4m writes), independently of the vector size. Considering the case of huge vector operations, in-memory associative processing eliminates the memory access costs and provides great performance advantage by its SIMD-like processing on each memory row.

### 2.2. Stencil Codes

**Figure 3:**
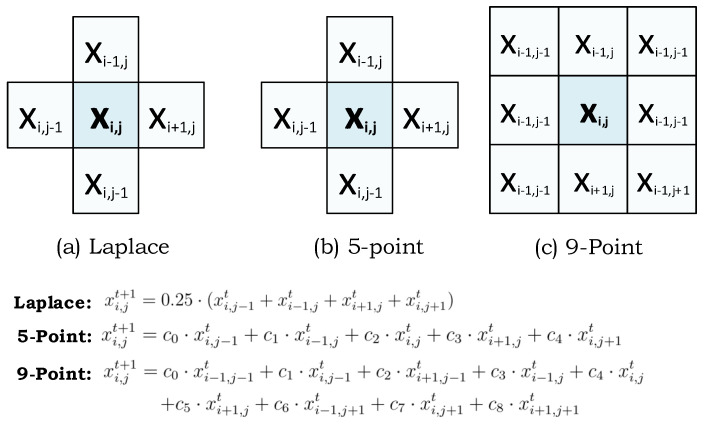
Three types of 2D stencil codes (Laplace, 5-point, and 9-point) together with their corresponding equations and computation patterns.

As introduced in the introduction, stencil codes are basic computational kernels that update an input array by following a specific pattern and mathematical equation. This update is performed over the whole array iteratively until a degree of convergence is obtained (e.g., a dependable weather prediction). The most common stencil type is the Laplace equation in which a cell is updated with respect to the average of its four neighboring cells. If the cell itself also included in averaging, the stencil code is named 5-point Jacobi iteration. The other stencil types are named as 7-point, 9-point, and 25-point, both on 2D or 3D data, which provides a weight for each cell by including more cells to the computation, respectively. Even though the figure presents three different types, there are many different types of kernels which perform different operations by following a different pattern like finite-difference time-domain (FDTD) stencil [39]. Depending on the shape of neighborhood cells, a different data processing application is obtained. In this way, the stencil codes can also be used for signal processing, especially on 2D image data. Figure 3 shows the three different stencil types with the visualization of their computation patterns and equations.

Even though computation seems trivial for stencil applications, memory bottleneck becomes a big problem since the computation is tightly coupled to the memory. Most stencil codes are categorized as memory-bound [29]; therefore, they suffer from memory bottleneck. Therefore, an efficient parallel implementation becomes very crucial. For this reason, GPUs are employed as the best processing environment until now rather than CPUs [5,26]. The reason is that even though GPUs have simpler and slower processing cores than CPUs, they can provide a high throughput to process such a huge amount of data since GPU cores have more memory bandwidth. When compared to GPUs, APs have much simpler cores, and its core density is huge (i.e., one memory row is like a processing core that can handle basic stencil computation) as presented in Section 2.1. Furthermore, AP performs the operations on the data directly, which virtually boosts memory bandwidth to memory size. At that point, a truly in-memory implementation of these applications on APs can provide more benefits than GPU-based implementations. Many studies in the literature prove that AP-based implementations of data-intensive applications have superior performance than the traditional correspondences [20,40,41,42,43], including the applications that has processing flow similar to stencil codes like fast Fourier Transform (FFT) [44]. Therefore, it is obvious that another memory-bound application of the stencil code can get a benefit, which is the main idea of this study. The following section presents the implementation in the AP in detail.

## 3. Accelerator Architecture for 2D Stencils

**Figure 4:**
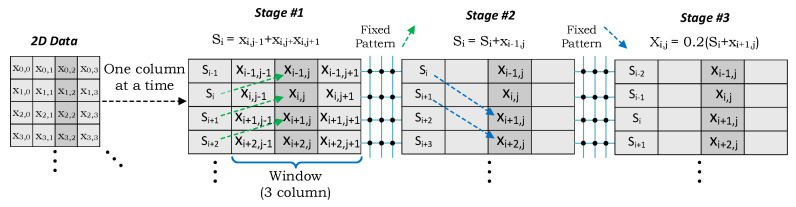
2D Stencil implementation (5-point iteration) on the associative processor (AP).

Figure 4 shows the proposed pipelined implementation of a 2D stencil (Laplace) in three AP stages where the data is transferred through the fixed interconnections between the APs. Each pipeline stage in the architecture performs the multiplication and addition operations with the corresponding neighboring cells and steers the data to the next stage. Since the communication pattern between the stages is known before, a fixed pattern can be defined in the circuit instead of having a configurable communication switch for which the area and energy costs are higher than the CAM array itself [19]. The pattern is the same for all three stencil types evaluated in this study (Figure 3).

In order to perform a stencil kernel on 2D data, data are sent to the accelerator as column-wise. This communication between the external memory (generally a DRAM) and AP can be handled by high speed dedicated buses so that CPU cycles are not wasted during the transmission. Each time, one column of the 2D data is placed to the AP sequentially starting from the first row. On the AP accelerator, the first stage keeps a three-column window inside to perform the stencil operation. The second and third stages also perform the addition operation between upper and lower neighboring cells together with the averaging operation to compute the final results as column-wise. For 5-point and 9-point stencil, these stages can also perform the multiplication operations with weights. On the other hand, the weight is generally set to get the average of these neighbor pixels. In that case, the operation can be converted to the sum of products, and multiplication operations can be performed in the last stage to get the average. In this case, a constant multiplication operation can be performed on all the rows for the faster and energy-efficient alternative. Compared to traditional CPU or GPU architectures, APs minimizes the memory access since data is moved once to the accelerator. Then the results are written back to the memory after an iteration. On the other hand, traditional architectures need to access memory whenever a cache miss occurs, so this leads to a huge number of circulation between the memory and the cache.

## 4. Evaluation

In the evaluation of proposed accelerator architectures, a cycle-accurate AP simulator is used, which can realistically perform the circuit simulations on Synopsys HSPICE in an iterative manner. For the transistors, 65nm predictive technology models are used [45]. For the memristor, a fabricated nano-second switching time device is referenced in the ReRAM-based AP architecture which has a size of 50 nm. Its corresponding SPICE model in [46] is used in the simulations. Since the AP supports fixed-point computation, data bitwidth is set as 32-bit. All data moving costs to the accelerator are taken into account as well as computation. For the stencil types, the multiplication constants are selected as equal (i.e., to perform the averaging operation). On the other hand, the architecture can support any type of numerical weights. In the circuit implementation, a CAM buffer is added in the first stage to increase the throughput so that during data movement, the computation can also be performed concurrently. Therefore, the total architecture consists of three computational stages and one buffer to receive the DRAM data. The following three subsections provide the details of the evaluation.

### 4.1. Fixed-Point Computation

**Figure 5:**
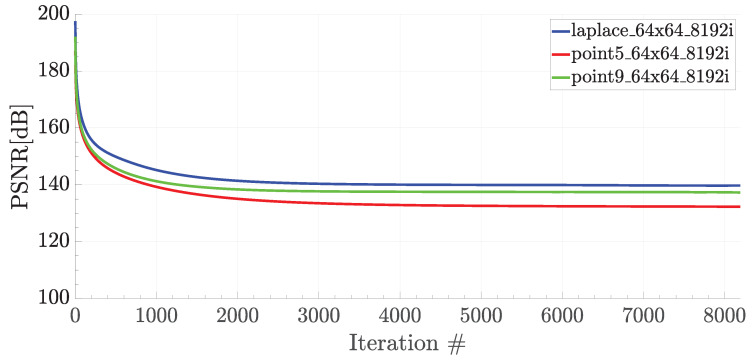
Peak signal-to-noise ratio (PSNR) with respect to the iteration number during various stencil operations on 64 × 64 matrices.

Due to the energy and performance issues of the traditional computer architectures performing on floating point, there is a trend towards using fixed-point architectures for the sake of performance and energy in the applications that can tailor some degree of inaccuracy, especially in the field of artificial intelligence and signal processing. Even some recent GPUs proposes a configurable precision architecture that can both perform operations on floating-point as well as fixed-point by delivering higher operations/second [47]. The stencil codes can also be evaluated under this class of applications, which can get benefit from the fixed-point computation. To evaluate this opportunity, a set of simulations were carried out in both floating-point (64-bit) and fixed point (32-bit). For the sake of simulation time, which takes more than one week for 256×256 matrix sizes, only 64×64 matrices were evaluated for three different stencil codes. Even though most FPGA/GPU-based stencil applications in the literature use floating-point arithmetic, our simulation results reported that 32-bit fixed-point calculation gave almost identical results to the 32-bit floating-point since the data were kept within a limited range during the stencil iteration (i.e., a kernel update includes averaging at the end). Figure 5 shows the peak signal-to-noise ratios (PSNRs) of three stencil codes over the iterations where the PSNR was computed with respect to double-precision (64-bit) floating-point. The value is computed as $\mathrm{PSNR}=10\;\cdot \;\log_{10}\left({\mathrm{peakval}}^{2}/\mathrm{MSE}\right)$, where the peakval is 1 for the normalized 64-bit floating-point numbers, and MSE corresponds to the mean-squared error between the fixed-point and floating-point results. Overall, the computation yields a high SNR rate of more than 100 dB. According to the results, the difference between the two computations was slightly increased over iterations (i.e., PSNR value decreased). On the other hand, the PSNR was settled down to its minimal value after some number of iterations. The results were very reasonable because the data kept in a limited range during the stencil. As an example, during weather prediction, the temperature range of the weather is generally limited in a range.

### 4.2. Comparison of Performance

**Figure 6:**
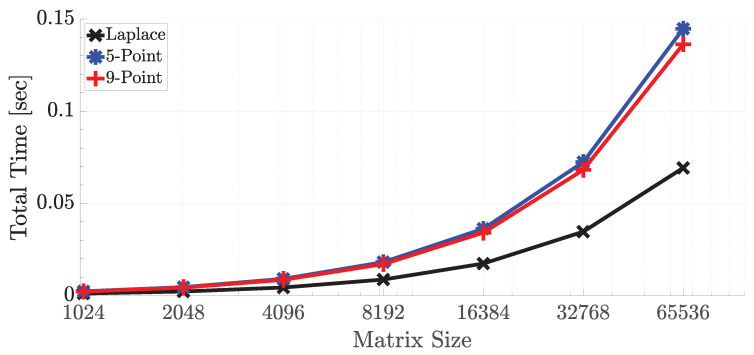
Single iteration run times of three stencil codes with variable array sizes of nxm where n is set as 4096 and m is between 1 K and 64 K.

**Figure 7:**
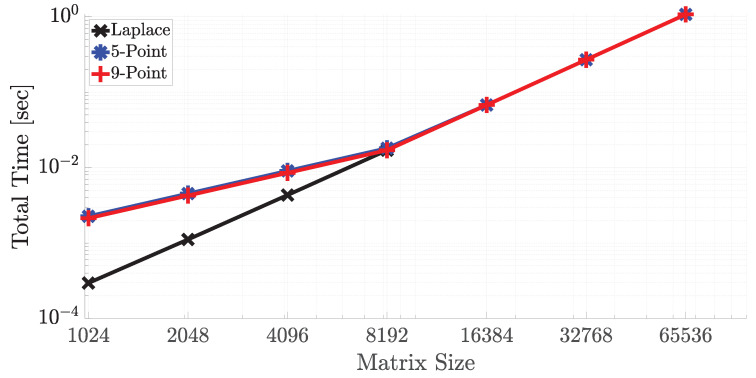
Single iteration run times of three stencil codes with variable array size of nxm where n = m.

The performance of the accelerator depends on some factors. If the array size is assumed as nxm where n is the number of rows and m is the number of columns, the run-time can be formalized as $\max(n\times t_{write},m\times t_{comp})$ where $t_{write}$ is the write speed to the CAM while reading data from DRAM and $t_{comp}$ is the total time that the slowest stage can finish its computation. As long as the array fits into the CAM as row-wise, $t_{comp}$ does not depend on the number of rows. It only depends on the bitwidth of the operands, and the next subsection on approximate computing presents the results on the effect of bitwidth. As stated in the previous section in which 32-bit fixed-point representation is enough for accurate computation, it is used to represent the numbers. Figure 6 shows the run time results of three stencil codes with variable matrix sizes on the SRAM-based architecture. Compared to SRAM-based architecture, ReRAM-based architecture had the same compare time, on the other hand, ReRAM requires two cycles for a write operation, and each write to ReRAM takes around one ns for the used memristor model. Therefore, ReRAM write operation was 4× slower than SRAM write. For this reason, ReRAM-based architecture was 50% slower. On the other hand, ReRAM-based implementation provided a 66% better area utilization in the memory area compared to SRAM-based cells since it was very compact and consists of two transistors and two memristors, where the memristor had a size of 50 nm. Furthermore, memristor-based implementation can facilitate probabilistic computing through its inherent stochasticity, which is a potential advantage over the traditional technology [48]. It is highly possible that it will come to prominence in the near future as the dark silicon area becomes more obvious [1]. In the results, the matrix size was selected as 4096 × m, where m changed between $2^{10}$ and $2^{16}$. As seen in the figure, Laplace transform took the least time since there was no required multiplication operation since the multiplication by 0.25 could be easily handled by shifting the point location in the number representation. For this reason, the data movement time dominated over the computation time. The 5-point and 9-point stencils gave almost the same results; however, the weight representation of 9-point stencil allowed faster multiplication since its binary representation had one less logic-1 compared to 5-point stencil. For them, the computation time dominated over communication time. On the other hand, if the total number of rows of the matrix exceeded 8 K, the communication cost dominated over computation, as presented in Figure 7.

### 4.3. Approximate Stencil Computing

Approximate computing is another promising approach for energy-efficient digital system designs, especially for error-tolerant applications like signal processing in the multimedia domain or neural networks [49]. In this approach, the accuracy requirement of the system is sacrificed at an acceptable level for the sake of performance and energy gains [50].

**Figure 8:**
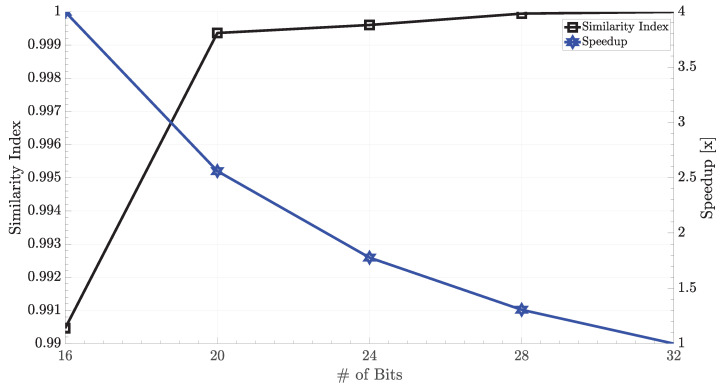
Results of approximate stencil code on the AP.

As stated in Section 2.1, an arithmetic operation can be started with any of the bits by disregarding their remaining right bits and go through the most significant bits since all operations are performed as bit-wise in the AP. For this reason, the associative computing provides a natural way of bit-wise dynamic approximate computing. Approximate computing is highly demanded, especially for signal processing applications to trade-off the accuracy for the sake of energy consumption and performance. In order to witness the effect of approximate in-memory computing on 2D stencil codes, the proposed accelerator was simulated under variable bit widths. Figure 8 shows the accuracy (i.e., similarity index) vs. speedup results with changing the number of bits. According to the results, 2.56× speedup was possible with an accuracy degradation of less than 1% when the bit width of operands was set to 20-bit instead of 32-bit. The speedup, in turn, provided more than 50% reduction in total energy consumption. This situation provided a perfect opportunity for edge devices at which power consumption was crucial. Compared to the traditional implementation of stencil codes on GPUs and FPGAs, the APs provided finer-grain reconfigurability for approximate computing.

## 5. Conclusions

This study shows a step towards solving the bottleneck problem in stencil applications through in-memory associative processing. The methodology mainly proposes combining the memory and CPU in the same place and exploiting each memory row as an individual CPU. To demonstrate this, a 2D stencil kernel is implemented in associate processors, and a comparison is made between the different stencil implementations. The results show that AP can provide an advantage for huge data amounts. Furthermore, the proposed methodology allows for bit-wise dynamic approximate computing, which is useful for signal processing applications. According to the results, the approximation at some reasonable level provides a considerable amount of energy savings and speedup in the system. Although the study focuses on stencil applications, it can be generalized to other signal and image processing applications on a massive amount of data such as convolution, filtering (edge detection, finite impulse response, etc.), and Fourier transform.

## Abbreviations

The following abbreviations are used in this manuscript:

AI	Artificial Intelligence
CPU	Central Processing Unit
GPU	Graphical Processing Unit
AP	Assciative Processor
CAM	Content Addressable Memory
FFT	Fast Fourier Transform
FPGA	Field Programmable Gate Arrays
PSNR	Peak Signal-to-Noise Ratio
MSE	Mean-squared Error
